# Supplementary material for: Community voices: Exploring beliefs, attitudes, practices and recommendations for improving stroke prevention and stroke care in rural and urban communities in Nigeria
Source: PLoS One. 2026 Feb 2;21(2):e0322157. doi: 10.1371/journal.pone.0322157 (PMC12863480; doi:10.1371/journal.pone.0322157)
Supplement: S1 Table — (DOCX) [file pone.0322157.s001.docx]

**Supplementary Data Information**

| **Table 1: Key Informant Results from Individual Interviews [Similarities and Differences] at the Rural (Ibarapa) and Urban (Ibadan) Sites Respectively** | | | | |
| --- | --- | --- | --- | --- |
| **Themes based on study Models** | **Rural Sites** | | **Urban Sites** | |
|  | **Orthodox Care Providers)** | **Unorthodox Providers** | **Orthodox Providers)** | **Unorthodox Providers** |
| *Risk susceptibility*   - *Risks/causes of stroke* | The dominant themes for **risks/causes of stroke in Ibarapa** KIIs center around: “*out of control blood pressure*; *God*; *evil people or witchcraft;* *unhealthy diets*; *use of alcohol*; while others reported “*bad air, lack or stressful exercise, smoking, older age, envy, drugs, lipids, snuf, stress, sugar diabetes or bad water* | | The dominant themes for **risks/causes of stroke** centered on *stress/strenuous jobs* and *heredity and/or blood pressure*. Other identified reasons include *diseases (heart, kidney)*; *lifestyle/sedentary lifestyle*; others that were identified included *bad air, alcohol, tobacco, evil people, herbs from alternative medicine providers, refusing treatment, obesity, older age, sin, state of the country* | |
| - *Do you think you are at risk of suffering a stroke in your lifetime?* | *Everyone is a potential candidate of stroke in our* *lifetime* ***(Medical Doctor – Ibarapa)***    *Well, it is not impossible. But I don’t think so because I check my BP, my lipids level, and my sugar level at least once in a year. And so, there is no fear at all* ***(Medical Doctor – Ibarapa)*** | ...*I don’t pray to have it because I have seen people suffer a stroke and the pain and agony is unbearable and that is why I prayed not to have a stroke* ***(Patent Medicine Vendor – Ibarapa)***  *I don’t think I am at risk because my blood pressure is always normal, and I don’t think of anything that can make me have any high blood pressure. I take this life very simple* (***Masseur, Ibarapa***) | *Well…I don’t pray to be part of the risk because I try as much as possible to prevent the risk….* Factors (causing stroke) include *hereditary, stress, BMI, salty diet, not eating enough vegetables and fruit, eating high quantity of food at a given time can make one have a stroke…alcohol…tobacco, also kidney disease, heart disease that are not well managed* (***Chief Nursing Officer – Ibadan***) | *There is nobody that cannot be a victim of anything* ***(Patent Medicine Vendor – Ibadan)*** |
| - *environment or health habits that can contribute to the providers’ risk of stroke* | *heredity* and/or *high blood pressure* were most common across both sites (n=8).  *Those that are obese, those with hypertension, high blood pressure without monitoring or treating it, diabetes, obesity; people that take alcohol, smokers, stressful jobs and do not take enough rest or concerned about their health* (***Matron - Ibarapa***)  …. *the market women, the elderly and even the youth, the young, the middle-aged people they are susceptible to stroke especially those that do not care about taking their blood pressure regularly and they have one ailment or the other and they refuse to take care for themselves* (***Chief Nursing Officer*** ***1 - Ibadan***) | | | |
|  | *Stress/strenuous jobs* was most common in Ibadan only  *Those who do strenuous jobs in our community just to get their daily needs but do not take care of themselves…… Youth of this age also take these drugs that empowers them and after which health problems come up in the future, Things like these causes what we are talking about* (***Patent Medicine Vendor - Ibadan)*** | | *God* was the second highest reason in Ibarapa (n=4) | |
| - *What do you fear most about stroke” and “how will you determine your risk/chances of having a stroke?* | Most all mentioned *paralysis*, *unable to get up or walk*, *confined to spot*, *incapacitated most of the time* and specifically followed by *turn a rich person into a poor person and the person will still die* (loss of income, death). Some included many fears: *anybody that has a stroke is liable to a lot of things. They depend on their families…. may not be able to move around…. need assistance and if family is not financially buoyant, they will just abandon them. Some can’t eat…. or go to toilet by themselves…. some will be dirty, stinking, and the rest*! Input from alternative/complementary medicine providers and healers also included *punishment from God* as a fear. Overall, their fears centered around *You can only manage it* (stroke), *it cannot be treated.* However, several Orthodox/Modern Medicine/ Health Care Providers noted that “*a few people recover but are at risk for a second stroke.* | | | |
| - *Who are more susceptible to having a stroke in their community, the reasons for their answers, and the effect of the environment or health habits that contribute to community members sufferings a stroke* | *There is no age range or limit to stroke but, in this community, I observed that men are mostly susceptible to stroke than women* ***(Physiotherapist - Ibarapa)***  *Smokers, alcoholic, fat people as it is common among them compared to the slim people, as the rate of blood flow is being elevated and when the sugar level is too high or too low can affect the brain, and when the brain is affected, It can lead to damage of the body system* ***(Masseur – Ibarapa)*** | *-* | *Well, within the community, let me say all ages because hypertension can affect all ages. Children can also have hypertension too* (***Chief Nursing Officer 2 - Ibadan***) | *Well, those who can easily develop stroke are the aged/elderly, they usually come down with it. People who are not too old now develop stroke too* (***Patent Medicine Vendor - Ibadan***)  *Our youths smoke and drink; they mix a lot of things with their drinks. This can cause stroke too…. Some people are too fat and have too much weight. Such people are supposed to check their BP all the time and limit their food and do a lot of exercise. If they fail to do that, they can have stroke* (***Patent Medicine Vendor 2 - Ibadan***) |
| **Risk severity**   - ***Please tell us what happens when someone has a stroke*** |  |  | *An individual that is suffering from a stroke, if you see the face, the face will not be normal; the mouth, a part of the lips may go down, the other part may be a little bit lifted up and they will not be able to talk very well* (***Chief Nursing Officer 2 - Ibadan***) | - |
| - **Why do you think a stroke starts when it does?** | *It may be as a result of family history, lifestyle, those less concerned with their health* ***(Matron – Ibarapa)*** |  | *You know we have different causes of high blood pressure it may be a clotted blood that is affecting a capillary or a vein, capillary in the brain and that capillary accumulated blood when there is no place to pass for the blood and it can rupture, and whenever a blood vessel or capillary rupture in the brain it causes stroke* ***(Chief Nursing Officer – Ibadan)*** | *Working without resting all the time and not taking care of one’s health or visiting the hospital for drug prescription* (***Patent Medicine Vendor - Ibadan*)** |
| - **What are the signs of a stroke prior to the medical diagnosis?** | *Someone whose brain is affected the part of the control the upper limbs are domicile could present with weakness or inability to move the upper limbs…So, they cannot swallow or speak well anymore. So, their voice changes…* ***(Medical doctor, Ibarapa)*** | *…it affects the leg and arms; there will be some weakness in some part of the body*  *eye get dimed and if he or she want to stand up it will be so discomfort for him to do that…Another sign and symptoms is that the blood pressure gets higher by the day. The heartbeat gets faster and make so much sound. These are the three signs and symptoms that shows prior to medical diagnosis (****Traditional bone setter 2 – Ibarapa)*** | *And one may not be able to walk, ….it will paralyze the legs; it will affect both or one, …., it will affect the tongue and one may not be able to talk very well; the speech is disturbed…The mouth may turn to one side; that person may not be able to lift up the hands, the legs too. And to walk, that person may not be able to walk. Then there may be incontinency of urine (****Chief Nursing Officer 1 – Ibadan****)* |  |
| - **What do you think a stroke does to the people in your community?** | *Stroke makes one financially deficient… they become dependent … You are the bread winner of the family; you are the providing the school fees of the family and suddenly you cannot do that again* ***(Physician, Ibarapa)*** | *-* |  | *It is a form of reproach to the person in the community. Assuming the person used to make good contributions to the community maybe in meetings, he can no longer do that because of his condition* (***Patent Medicine Vendor - Ibadan***)  …*it makes them not to be able to work again, to provide for their family* (***Patent Medicine Vendor 2 - Ibadan***) |
| - **How severe are most strokes? Do they have a long or short course?** | ***-*** | *…That of been bewitched, when it befalls someone, such person can be bedridden for ten years, with inability to walk and will eventually die. And for some people, they can only last 5 days to 3 days, and they are dead* ***(Traditional healer – Ibarapa)*** | *Hmmn...* *Most of them are bad so that before they can come out of it, it can take a long time. Some people may not come out of it and eventually they may die. Some of the stroke last forever, and sometimes anyway the prognosis is bad* (***Chief Nursing Officer 1 - Ibadan***) | - |
| - **What kind of treatment do you think people who have a stroke should receive?** | *…intensive care unit have to observe the patient... Sometimes surgeons can come in... On the long way the physiotherapy will need to come in for rehabilitation and another specialist too must come in…It’s always a multi-specialty approach…* ***(Doctor, Ibarapa)*** | *It could be either modern medicine or traditional medicine. But the most important is early presentation of the ailment. Both treatment are good. The kind of recommendation that I can outline include going to hospital regularly. The reason why I said hospital is they will quickly know if your health status is good or not. Early presentation by the patient is the key. When they go early to the to the hospital, the doctor will know whether there are other disease within the system of patient.* ***(Traditional bone setter 2 – Ibarapa)*** | *Whenever they bring the patient to the hospital, they will give drugs, …. they will invite specialist like counsellor, health educators, which I am one of them to counsel the patient to modify the lifestyle of the patient ……we will invite the physiotherapist to work on the patient on active and passive exercise*” (***Chief Nursing Officer 2 - Ibadan***)  “*When the sufferer comes to the hospital, they will check the blood pressure, ……Different tests and diagnosis will be carried out to know the cause of the stroke and possible treatments* (***Matron - Ibadan***) | *-* |
| - **What are the chief problems that a stroke causes for persons and their families in your community?** | *It is a burden for the family and for the sufferer it is a burden on its own, there will be stress and lethargy, and such will be useless for himself.* ***(Masseur – Ibarapa)*** | - | *Most of them are just managing themselves. So the family members will be stressed trying to get funds to take care of the patient. Especially if the patient is not on health insurance the family member will have to source for funds to take care* (***Chief Nursing Officer 1 - Ibadan***)  *For the family, if the person is the bread winner, he will not be bringing money again* (***Chief Nursing Officer 2 - Ibadan***) | *-* |
| - **What do the people in your community fear most about a stroke?** | *…they don’t what to wake up with stroke… They don’t want to hear about it … And they know that it comes with a capital-intensive treatment …it involves a lot of money and stroke treatment takes almost of their money…* ***(Medical doctor – Ibarapa)*** | *What I fear most about the ailment it is that it does not allow the patient and the family to have rest. It is an ailment that takes most of the relative time and money consuming. A sickness that come and never heal easily. Even most time, the patient does not get heal and eventually dies.* ***(Traditional bone setter 2 – Ibarapa)*** | *It may lead to death of that person that have stroke. Then some feels it’s a stigma that is going to be a stigma on that person that people would not like to get closer. Another is how to take care of them which is the most essential thing* (***Chief Nursing Officer 1 - Ibadan***) | *What they fear most is seeing a once active man who goes up and down for journey, business and trade but is now bed-ridden, fed and taken to the toilet. They are afraid of this condition, and they say it within the community that the person has been knocked down by stroke* (***Patent Medicine Vendor - Ibadan***) |
| *Benefits to Action-*   - *What different actions do people in your community take when they have a stroke?* | *Some will seek for orthodox care in the hospital, some go to herbalist (non – orthodox) to give them non-orthodox drugs to rub on their hands, some give them incantations. Then majorly they seek non – orthodox more than orthodox* ***(Matron – Ibarapa)*** | *…There is this OPC man who had issue, we all knew what happened to him was beyond the natural, he was taken to the hospital and was told he needed surgery, when I heard it, I told them that he will get well without surgery….but the bottom line is that they involved me.* ***(Traditional healer – Ibadan)***  *They sometimes take them to the traditional healers such as herbalists, witch doctors etc.* ***(Patent Medicine Vendor – Ibarapa)*** | …. *they believe in self-medication. They will not bring themselves to the appropriate place* (***Chief Nursing Officer 1 - Ibadan***) | *According to our tradition, there are some cases that are taken care of through non-orthodox medicine…*  *We do not know anything about the herbal medicine, but the family members are the ones who go that way after getting advice from neighbors and people within the community…The children, wife, and family members will take the person to the hospital for treatment before returning the person home for continuous care*….*Once they see that someone has developed stroke, they will go and buy this balm (name not mentioned), boil snails quickly and eat it. Different types of advice you will receive. Some followed the advice and got well* (***Patent Medicine Vendor - Ibadan***) |
| - **What are the benefits of each of the actions that you have described?** | *No benefit but they hardly believe that there is a cure or there could be improvement when they go for medical treatment, exercise, and physiotherapy* ***(Masseur – Ibarapa)*** |  | *It helps a lot because it will prevent further damage and prevents death* (***Chief Nursing Officer 2 - Ibadan***)  *Whoever comes to the hospital immediately stroke happens, it is certain that such person will recover on time more than those that went to church, mosque, or traditional herbalist because when the cause is diagnosed on time, the high blood pressure will come down, the diabetes will reduce, the sickness will subside* (***Matron - Ibadan***) | *Benefits* *are for the stroke patient to get better and survive* (***Patent Medicine Vendor - Ibadan***) |
| - **What actions do you recommend that a person with a stroke take? What are the benefits of those actions?** | *With a stroke, well my recommendation is this once you have a stroke already, they should be close to a doctor, be close to your hospital* ***(Medical doctor – Ibarapa)***  *For someone that has stroke, it is easy now as we have physiotherapy, massaging machine and regular medications etc. can be of help.* ***(Masseur – Ibarapa)*** | *I will recommend hospital treatment* ***(Patent Medicine Vendor – Ibarapa)*** | …*is going for appointment, not missing medical appointment, follow up, exercise, physiotherapy, diet, relieving everything that can make one to be anxious or that can cause stress. Then stopping tobacco or alcohol smoking, eating more of fruits and vegetable especially when one is above forty years of age, low salt and Maggie diet* (***Chief Nursing Officer 1 -- Ibadan***)  *Then whenever they develop stroke like this, bring the patient to the hospital straight away* (***Chief Nursing Officer 2 - Ibadan***)  *For anyone who has a stroke, it’s better to go to the hospital first*… (***Matron - Ibadan***) | *Regular exercise with the machine they usually use for them. If such a person can afford to buy the machine it will be very good. Also, taking to the medications given can help the person recover* ***(Patent Medicine Vendor – Ibadan)*** |
| - **Are most people able to take those actions you recommend and why or why not?** | *Yes, they do take this advice most especially elites, and those who have the educated ones around them. For those who don’t, they believe they are spiritually cursed and if such a person was a former traditional worshipper, so he goes to appease their ancestors or gods*  ***(Masseur – Ibarapa)*** | *Yes, they do. In fact, in my community, they do. I do tell them not to overtake the herbs, not to womanize because it won't work for them. We tell some to be watchful of what they eat and they do. They all follow instructions* ***(Non-orthodox doctor – Ibarapa)*** | *The people in the community are ready to get information and are ready to make use of those information so far that they know that it will have positive impact on their health* (***Chief Nursing Officer 1 - Ibadan***)  *Most people are not able to take those actions because of financial problems. Many don’t have the money* (***Chief Nursing Officer 1 - Ibadan***)  *And many may be afraid of what the sickness may lead to. Many may take it that that is their own destiny, that if death comes it’s okay* (***Chief Nursing Officer 1 - Ibadan***) | “*They took the recommended action because I follow them up and they do come here a times to get the drugs prescribed for them. Most people don’t go to the hospital because they know that they will do tests, buy drugs, and pay bills and they don’t have the money. Most of these people can’t afford to eat. There’s no way they can go to the hospital*.” (***Patent Medicine Vendor 2 - Ibadan***) |
| *Barriers to action*   - **Barriers or reasons that interfere with people following your recommended actions** | *Some of them will say they do not have money, while some do not have interest in orthodox drugs, as they are of the school of thought that drugs will only suppress it and not necessarily cure it.* ***(Matron – Ibarapa)*** | *Nothing hinders them more than poverty because they know they can’t go to the hospital without spending money and so resolve to feeding themselves with what they have and when death comes, that’s it* ***(Masseur – Ibarapa)*** | *People are afraid of hospitals; they don’t want to pass through any rigorous action in the hospital. Any stress in the hospital they don’t like it. …. They want quick things. …… so they want fast action, they want fast intervention and it is not possible* (***Chief Nursing Officer 2 - Ibadan***)  *They may give excuse on the proximity of the hospital to their house* (***Matron - Ibadan***) | *The first one is money. Once we advise them to take the person to the hospital, they will tell us that it will gulp money as treatment is not free in the hospital. Another problem is that they will say once they take the person to the hospital, they will prescribe many drugs that will be expensive to buy. They might even tell them to buy walking stick for the patient but if there is no money, they will not do it. That is why they end up saying they should take such a person to the herbalist and see how the treatment goes* (***Patent Medicine Vendor - Ibadan***)  *What can hinder my belief is that when a doctor in UCH embark on strike and the hospital is shutdown, ……… When you become disappointed in a system you hold in high esteem, then your faith in them also diminishes too* (***Patent Medicine Vendor - Ibadan***) |
| *Self-efficacy (SE)*   - **Community’s experiences with stroke** | - | - | - | *The arm or leg will lose balance, the mouth shifts to one side, inability to speak. We see a lot of people with speech impairment. It also comes with paralysis of the left or right body parts. The common signs now are loss of voice, that they cannot even say what is happening to them. Some comes with hearing impairment, and I have three to four persons with such in my community* (***Patent Medicine Vendor - Ibadan***)  *I know of just 2 cases in this community….They are getting better. The man can walk around now, and he can use the hand, the other person it affects her speech, and she too is getting better now*…. (***Patent Medicine Vendor 2 - Ibadan***) |
| - **Describe what happens to the person when they have a stroke—what changes physically?** | - | - | *The inability to walk with the paralysed limbs; the mouth turning to one side* (***Chief Nursing Officer 1 - Ibadan***) | *In some people it may affect one side of the body, one hand and one leg. To some people it may affects their speech and makes them stammer when trying to talk* (***Patent Medicine Vendor 2 - Ibadan***) |
| - **Who talks to people who had had a stroke and what do they recommend that the person does?** | - | - | - | *Once we tell them, they are ready to follow it, because they trust us. Someone like me, a traditional healer in this environment, they trust me. I am the chairman of the traditional healers, I am also a community leader. So when we are having meetings, I do sensitize them* (***Patent Medicine Vendor - Ibadan***) |
| *Cues to action*   - **Cues or reasons that trigger people to accept a recommended health action for preventing a stroke?** | *…It depends on the approach of the caregiver, and when they have examples of those that have had it before and recovered.* ***(Masseur – Ibarapa)*** | - | ...*some believe if they strictly adhere to what you tell them for the prevention of not having a stroke, some believe that it will help them a lot. So they adhere to your recommendations, to your health education and many don’t want to die, many don’t want to be confined to an area for so long so whatever you tell them not to do, many take to it* (***Chief Nursing Officer 1 - Ibadan***) | - |
| - **How costs influence a person’s or family’s or community actions for prevention** | - | *Let’s say the family of the patient take him to a general hospital and the advice that the patient need to be admitted. Without money nothing can be done. They either abandon the patient there or they run away* ***(Traditional bone setter 2 – Ibarapa)*** | *The person or the family member may not be able to afford prescribed drugs that may help reduce high blood pressure, diabetes or obesity* (***Matron - Ibadan***) | - |
| - **Cues or reasons that trigger people to accept a recommended health action for treating a stroke** | *Poverty because many complain of not having money and some will say, they can’t spend money on buying drugs on a daily basis.* ***(Masseur – Ibarapa)*** | *If somebody have stroke and he was asked to buy honey, food or to buy what we will use for him if there is money to buy those things there is nothing we can do…* ***(Traditional Bone setter – Ibarapa)*** | *If there is insurance scheme; if they know that if they go to the hospital they are going to pay less* (***Chief Nursing Officer 1 - Ibadan***)  *The attitude of health care personnel* (***Chief Nursing Officer 1 - Ibadan***) | - |
| - **How costs influence person or family or community actions for treatment and recovery or management of stroke** | - | - | *Anything they want to do they have to pay. No free medical treatment, nothing is being supplied. As they go from one stage to the other, they have to pay. If money is not readily available to make the payment it will hamper the treatment that he is supposed to have at that particular time. The patient that have the stroke will not recover on time* (***Chief Nursing Officer*** ***1 - Ibadan***)  “...*maybe the person has the brother or sister or the family that are contributing for her hospital fee or for the drug. If they stop giving her the money, it is where they stop that the treatment will stop.”* (***Chief Nursing Officer* 2 - Ibadan)**  “… *the cost of treating a stroke is more than the cost involve in preventing it. Therefore, when there is no financial assistance, recovery may be difficult and it will affect the family because a family member will be required to take care of the person with stroke, feed and cater for the family members.”* (***Matron - Ibadan***) | *People that have stroke have to remain on drugs for the rest of their life and they have to eat fruits. These will cost them money* (***Patent Medicine Vendor*** ***2 - Ibadan***) |
| **Recommendations for improving Stroke prevention and care**   - **How the University of Ibadan can help to improve stroke prevention in your community** | *They can sponsor a billboard and posters to make enlightenment, help them to equip health centers ehm with sophisticated facilities for diagnosis and treatment. Also, then human resources for instance in this Ibarapa we don’t have any center for diagnosis so if there have health center with human resources and material resources are put in place so that if we have a stroke patient, they will be well taking care of* ***(Medical doctor, Ibarapa)*** | - | *The way it can help is if they can do awareness on television, radio for people to know in their language that these are the things that can cause stroke. Then if they can fund outreaches or medical treatment for people* (***Chief Nursing Officer***­ ***1 - Ibadan***)  ...w*e are going to give more education, … we are planning to go to the community for blood pressure, checking of blood pressure is a great thing. So it will surely help the community* (***Chief Nursing Officer 2 - Ibadan***) | …*do community outreaches regularly with government support where you organize health education for the people… people will know the causes and how to prevent it. If you create awareness on the causes like smoking, taking alcohol and drugs, these behaviors will reduce, and it will reduce the number of stroke cases* (***Patent Medicine Vendor - Ibadan***)  *People that have stroke have to remain on drugs for the rest of their life and they have to eat fruits. These will cost them money* (***Patent Medicine Vendor*** ***2 - Ibadan***) |
| - **How can the University of Ibadan help to improve care for persons who have a stroke in your community?** | *Okay ehm also providing provisions of sophisticated equipment because after you have dedicated people then you should be able to give them what they will work with, there is no point in dedicating without something to work with. That means you need to provide the equipment and all that* ***(Medical doctor, Ibarapa)*** | *If the project can give some free treatment to the sufferers for example. Then on the issue of treatment, it can be made free, occasional visits, and motivating them in form of tokens to be given to them.* ***(Masseur – Ibarapa)*** | …*assist in the payment, in providing treatment; help them with subsidy for the investigation and the medication* (***Chief Nursing Officer*** ***1 - Ibadan***)  ..*at least once in a week they must come and check their blood pressure, and then we do call them: how are you feeling, hope you are using your drug? we health-educate them on their diet and some bad habit, and there is a form that has been giving to them we ask them questions about their well-being* (***Chief Nursing Officer*** ***1 - Ibadan***)  *Providing financial help can help reduce the burden of the cost on the person suffering from stroke …by bearing the larger part of the cost, half or one-third of the cost implications for their treatm*ent (***Matron - Ibadan***) | *They can be providing free treatment to those who have suffered the attack, and they should also be going for mobile counselling* (***Patent Medicine Vendor - Ibadan***)  *My first recommendation: go to community health center and mobilize to come check their blood pressure, blood sugar, if you can afford wider test, malaria parasite. …... With these series of investigations people will know their limit of their BP and it will be followed by proper counselling* (***Patent Medicine Vendor - Ibadan***) |
| - **Recommendations for improving the SISS approach** | *…we can update them with education and information regularly. We can also print pamphlets, fliers, stickers and posters to project this* ***(Masseur – Ibarapa)*** | *provide lines that people can get access to them. I mean phone number and if it is company that is in charge of the program, phone lines should be made available. For example, if they have a stroke patient, they can call ARISES that how can they be of help for this type of people (****Traditional bone setter 2 – Ibarapa)*** | …. *hot line that they can call free of charge* (***Chief Nursing Officer*** 1 - ***Ibadan***)  *If you can give them free drugs, some may say they don’t have money to buy their drugs. So as you said calling them often to know about their conditions, then visiting them …….to know about how the family members are caring for them - those are my recommendations* (***Chief Nursing Officer 1 - Ibadan***) | *Establish a center in this community, where people can go* (***Patent Medicine Vendor - Ibadan***) |
