# Supplementary material for: Community voices: Exploring beliefs, attitudes, practices and recommendations for improving stroke prevention and stroke care in rural and urban communities in Nigeria
Source: PLoS One. 2026 Feb 2;21(2):e0322157. doi: 10.1371/journal.pone.0322157 (PMC12863480; doi:10.1371/journal.pone.0322157)
Supplement: S2 Table — (DOCX) [file pone.0322157.s002.docx]

| **Table 2: Focus Group Results from Community Leaders and Members in Ibadan and Ibarapa** | |
| --- | --- |
| *Risk susceptibility*— | The FGDs were asked to identify risks of stroke in the lifetime of persons (and comments are listed as examples of most to less common risks across the FGDs):   - *We are all in danger of having a stroke. No one knows what causes stroke, maybe it’s from wind, maybe it is from water….so we are in danger, and we do not have rest of mind, because no one know who it can affect. May God no let bad things happen to us and those it has happen to, may he heal them.* - *Many things can cause the sickness.* - *If one loses a child and one thinks of it out of proportion, it can cause hypertension which will lead to stroke.* - *If our surrounding is dirty, it can also cause this sickness (stroke).* - *Injection; the immunization that is being given to children can cause stroke….* - *What I think is the cause….is the state of the country. There is no rest of the mind…* - *bananas/carbide, bad water, Maggi, Lacasera, 7-up.* |
| *Risk severity* | The groups were asked to describe a)what happens when someone has a stroke, b) why it starts when it does, c) the signs of stroke, d) what it does to people in the community, the severity and length of time, as well as treatment the stroke patient should receive, the chief problems that a stroke causes for the person and their families, as well as what they fear most about a stroke. Their replies included:   - *Many people are running away in the sense that if someone is afflicted with stroke, they might say it is one woman that caused it or they might say witches are too many in a certain family.* |
| *Benefits to action* | The different actions people in the community take when they have a stroke & why they take the actions as well as actions the FGDs would recommend that a person with a stroke take, the benefits of those actions, and if most people were able to take those recommended actions and why or why not. FGD replies included:   - *The procedure that can be laid down for stroke sickness are many; first, we may tell him that this thing that happened to him, he should use traditional cure like herb for it. He should be drinking herbal drinks, if it then shows that it is not responding as expected, they will now tell him to go to hospital for modern treatment.* - *If he sees people that will help, that will support him in terms of treatment, he will go to the hospital on time, where he will receive treatment and there will find a way out. The kind of treatment he supposed to receive, our government supposed to have a specialized hospital….* - *The help we can render is to call those that are specialists (on stroke) to come over to our area and give lectures of what can cause stroke sickness and ways to curb it. We should be telling people around us about the danger in having stroke, more so, they should always be taking care of their body.* |
| *Self-efficacy and Cues to Action* | To explore self-efficacy and cues to action, the FGDs were asked to: a) identify and share their beliefs and beliefs from members of their community (about ways their capacity to act in the ways to prevent/improve stroke outcomes) b) identify cues or reasons that trigger people in their community to accept a recommended health action for preventing a stroke and c) who makes these recommended health actions and their replies included::   - *“I am ready to change my behavior concerning health and what I eat.”* - *“We can go to the stroke specialists to talk to them that they need to come and give lecture to our people in that area.”* - *“Money has impact on stroke. If one has money, he can receive treatment anywhere he feels like but if God says it is the sickness that will kill him, there is nothing anybody can do.”* - *“They (stroke specialists) make research on what causes stroke.”* |
| General comments/  recommendations  for working effectively with communities | The FGD leaders also shared that ARISES has recently started to work on a system for tracking stroke in their community (SISS). However, the ARISES team needs to know:   1. What are your recommendations for improving the approach? *(*The FGD leader also shared the realities related to funding: ***Please realize that we may not be able to follow your instructions if it requires funding that we cannot support but it will give us additional ideas for future work).*** 2. What else would you like to tell us about stroke and what your community needs to help improve stroke?   The FGD participant messages include:   - *“ARISES should form representatives to visit rural areas because everything we are doing here is not known to people in the rural areas. Some people should be sent to them to lecture them in rural areas from one vicinity to another. This will also be helpful apart from phone.”* - *“On the issue of preventing stroke, ARISES, advice government on what they should be using which will not pose a problem to them. Government has the go-ahead if stroke will be reduced. It cannot be totally eradicated but it can be reduced.”* |
