# Supplementary material for: Community voices: Exploring beliefs, attitudes, practices and recommendations for improving stroke prevention and stroke care in rural and urban communities in Nigeria
Source: PLoS One. 2026 Feb 2;21(2):e0322157. doi: 10.1371/journal.pone.0322157 (PMC12863480; doi:10.1371/journal.pone.0322157)
Supplement: Attachment — Submitted filename: Reviwer comments.docx [file pone.0322157.s003.docx]

Title: **Community Voices: Exploring Beliefs, Attitudes, Practices and Recommendations for Improving Stroke Prevention and Stroke Care in Rural and Urban Communities in**

**Nigeria**

Comments:

This is an important qualitative study which explores and contrasts the topic of stroke from alternative and modern medicine perspectives, but at the same time looks at rural and urban perceptions. By doing this, the author is able to comprehensively tackle the topic and bring out all the important issues around beliefs, attitudes, practices and recommendations for improving prevention and care for stroke in a low resource setting.

Unfortunately, the author needs to work on the manuscript to make it a smooth read for publication.

1. The introduction in the abstract and the manuscript should be tailored towards the topic more than constant reference to the research team/group (ARISES)
2. The same applies to the method section which is filled with descriptions about the research team/group and not much detail about recruitment criteria and sampling. Was the sampling method the same in rural vs. urban? alternative vs. modern medicine? The 5 persons who refused to participate due to conflict of time, which group where they from?
3. There are several published works which have touched on one aspect or the other of this research work. Therefore, the author might have to rephrase on this paper being the first to investigate and contrast community beliefs........
4. The results section could have been better reported by focusing on comparisons e.g. rural vs. urban and alternative vs. modern medicine in table form as opposed to tables 3 and 4, which are lengthy and difficult to follow. The recommendations for improving prevention and care for stroke patients could also have been presented in table form.
5. The results section could also have clearly outlined the similarities and differences between rural vs. urban findings with regards to alternative vs. modern medicine for stroke prevention and care.
6. The author should also work on the typographical and grammatical errors in the manuscript.

All in all, this is a good manuscript which makes a significant contribution to the community voice and can be improved for publication.
