# Supplementary material for: Community voices: Exploring beliefs, attitudes, practices and recommendations for improving stroke prevention and stroke care in rural and urban communities in Nigeria
Source: PLoS One. 2026 Feb 2;21(2):e0322157. doi: 10.1371/journal.pone.0322157 (PMC12863480; doi:10.1371/journal.pone.0322157)
Supplement: Attachment — Submitted filename: RESPONSE_TO_REVIEWERS_ARISES_PLOSONE_16122024.docx [file pone.0322157.s005.docx]

**ARISES MANUSCRIPT**

**RESPONSE TO REVIEWERS**

16^th^ December 2024

Dear Editor-in-Chief,

Thank you for your careful attention in providing feedback on our manuscript. Per the recent comments raised [with resubmission], please find below my responses (*in italics*) for each of the items flagged:

1. PLOS requires an ORCID ID for the corresponding author in Editorial Manager on papers submitted after December 6th, 2016. Please ensure that you have an ORCID iD and that it is validated in Editorial Manager. *This has been resolved with the recent link of my ORCID number along with the online resubmission platform.*
2. In the online submission form, you indicated that [Data will be available upon request from the author as data contains recorded voices.]. All PLOS journals now require all data underlying the findings described in their manuscript to be freely available to other researchers, 1. In a public repository, 2. Within the manuscript itself, or 3. Uploaded as supplementary information. This policy applies to all data except where public deposition would breach compliance with the protocol approved by your research ethics board. If your data cannot be made publicly available for ethical or legal reasons (e.g., public availability would compromise patient privacy), please explain your reasons on resubmission and your exemption request will be escalated for approval.

*Thank you for your comment and apologies for lack of clarity in our earlier responses. We have now gone back to check our previous responses obtained by our participants as approved by our Ethics Board. Per the conditions of the informed consent document, the study team destroyed audio recordings after verifying the accuracy of the transcripts. Also, the informed consent signed by participants stated that the study team would employ all means possible to maintain the confidentiality of their personal information and that data collected would be used solely for the purposes of this study, with access limited to authorized study personnel only.*

3. We note that you have indicated that there are restrictions to data sharing for this study. PLOS only allows data to be available upon request if there are legal or ethical restrictions on sharing data publicly. For more information on unacceptable data access restrictions, please see http://journals.plos.org/plosone/s/data-availability#loc-unacceptable-data-access-restrictions.

Before we proceed with your manuscript, please address the following prompts: a) If there are ethical or legal restrictions on sharing a de-identified data set, please explain them in detail (e.g., data contain potentially identifying or sensitive patient information, data are owned by a third-party organization, etc.) and who has imposed them (e.g., a Research Ethics Committee or Institutional Review Board, etc.). Please also provide contact information for a data access committee, ethics committee, or other institutional body to which data requests may be sent.

*Thank you for your comment and apologies for lack of clarity in our earlier responses. We have now gone back to check our previous responses obtained by our participants as approved by our Ethics Board. Per the conditions of the informed consent document, the study team destroyed audio recordings after verifying the accuracy of the transcripts. Also, the informed consent signed by participants stated that the study team would employ all means possible to maintain the confidentiality of their personal information and that data collected would be used solely for the purposes of this study, with access limited to authorized study personnel only.*

b). If there are no restrictions, please upload the minimal anonymized data set necessary to replicate your study findings to a stable, public repository and provide us with the relevant URLs, DOIs, or accession numbers. For a list of recommended repositories, please see

https://journals.plos.org/plosone/s/recommended-repositories. You also have the option of uploading the data as Supporting Information files, but we would recommend depositing data directly to a data repository if possible. We will update your Data Availability statement on your behalf to reflect the information you provide.

*Thank you for your comment and apologies for lack of clarity in our earlier responses. We have now gone back to check our previous responses obtained by our participants as approved by our Ethics Board. Per the conditions of the informed consent document, the study team destroyed audio recordings after verifying the accuracy of the transcripts. Also, the informed consent signed by participants stated that the study team would employ all means possible to maintain the confidentiality of their personal information and that data collected would be used solely for the purposes of this study, with access limited to authorized study personnel only.*

4. Concerns have been raised that this manuscript is very closely related to the following papers, of which you are an author: Jenkins C, Ovbiagele B, Arulogun O, Singh A, Calys-Tagoe B, Akinyemi R, et al. (2018) Knowledge, attitudes and practices related to stroke in Ghana and Nigeria: A SIREN call to action. PLoS ONE 13(11): e0206548. https://doi.org/10.1371/journal.pone.0206548

Our second publication criterion notes that "If a submitted study replicates or is very similar to previous work, authors must provide a sound scientific rationale for the submitted work and clearly reference and discuss the existing literature. Submissions that replicate or are derivative of existing work will likely be rejected if authors do not provide adequate justification." Please see http://www.plosone.org/static/publication.action#results for more information.

Please provide a clear rationale for the necessity of the study in this submission. Please also explain how the work described in this submission differs from and/or advances on that described in the related paper, and which findings can be considered unique to your submitted manuscript.

*Thank you for your comment. Similarities and differences relating to the above studies have been expatiated on Table 1.0 below.*

| **TABLE 1.0: SUMMARY OF PLOS ONE QUALITATIVE RESEARCH MANUSCRIPTS –**  SIREN [JENKINS ET AL 2018] AND ARISES QUALITATIVE MANUSCRIPT (2024) | | | |
| --- | --- | --- | --- |
| **SN** | **DIFFERENCES IN THEMES ACROSS PAPERS** | **SIREN [JENKINS ET AL, 2018 PAPER]** | **ARISES CURRENT MANUSCRIPT** |
| 1. | Study objective | To **highlight the attitudes, beliefs and practices relating to stroke** and to propose future approaches for implementing community based participatory research including participation and acceptance of genomic studies and genetic testing respectively. | To describe the **differences** in beliefs, attitudes, and practices related to stroke risks, prevention, and care delivery in Nigeria. **S**pecifically, it aimed at **designing the SISS by unravelling the barriers and facilitators recognition, early presentation and care of stroke patients in urban versus rural settings in Nigeria.** |
| 2. | Study sites | 15 sites in Nigeria and Ghana | 2 sites [rural & urban] in Southwest Nigeria |
| 3. | Study setting | SIREN is Hospital based. | ARISES is **community/population** based which compared findings across the above sites. |
| 4. | Study participants | Stroke survivors, caregivers, health professionals, religious and community leaders | **Alternative/complementary providers/healers,** orthodox/modern medicine/health care providers [Medical Doctors, Physician Assistants, Neuroprofessionals, Registered Nurses, community leaders [including religious **(faith-based), traditional leaders]** |
| 5. | Qualitative research approach | Focus Group Discussion (FGD) | Focus Group Discussions (FGDs) & **Key Informant Interviews (KIIs)** |
| 6. | Study findings | Findings revolved around stroke-related fears, causes, associated problems, how to access treatment, recommendations on how to work with the SIREN sites in addressing these concerns ~~whilst~~ while increasing acceptability and participation in genomic studies. | Per its findings, this study identified varying risk factors for stroke including environmental, lifestyle, hereditary and spiritual causes [God, witchcraft, evil etc]; stroke related fears and problems – both for the stroke sufferer, family and community; barries to uptake of recommended preventive measures, suggested ways to improve stroke care and prevention across communities including collaboration between orthodox and unorthodox/alternative/complementary providers. |
| 7. | **Study limitations** | | **Gaps addressed by ARISES study** |
|  | First, the findings from the study were grouped [lumped together]. The danger in adopting this approach is that each community is peculiar in context, cultural values and setting. Their individual peculiarities may be submerged, thus masking access to relevant data required to guide implementation of context-specific interventions for greatest positive impact and outcome within each site. | | Data obtained from this study was ‘ungrouped’. Findings were based on each study location [urban and rural setting] and is a representation of the beliefs, practices and attitudes of each community. Thus, each communities’ findings is key in guiding design, implementation of context-relevant interventions for the greatest impact per community. |
|  | Secondly, in-depth interviews were not conducted because of budgetary constraints. IDIs, as part of qualitative research, are highly needed to provide the much-needed data (until saturation) for a more robust and comprehensive insight on the concept of beliefs, attitudes and practices. | | To ensure data saturation on the topic, twenty-four (24) KIIs were conducted, this time, with alternative/complementary providers/healers, orthodox/modern medicine/health care providers at each study location. This provided further insights to challenges for accessing orthodox/modern health care providers (HCP) which ultimately are drivers towards patronage of alternative/complementary providers/healers at both communities. |
|  | Thirdly, though religious leaders were part of the participants, not much data on the impact of religion and its influence on stroke-related beliefs was elucidated in this study. | | Having identified the adoption of alternative/complementary providers/healers as alternate treatment option under the SIREN study, engaged and explored in detail, alternative/complementary providers/healers via KII sessions in a bid to highlight their beliefs, attitudes and practices in stroke prevention, care and recovery. |
|  | Fourth, consultation of alternative/complementary/non-orthodox providers and faith healers was not given deep exploration despite being identified as alternate treatment options. | | ARISES paper brought to the fore, an interesting finding which is the need for a collaborative partnership between alternative/complementary providers/healers and orthodox/modern medicine/health care providers with the sole aim of closing the gap in provision of stroke prevention, care and rehabilitative services which will ultimately improve quality of life especially for stroke sufferers. |
|  | Some SIREN study participants were recruited from health facilities, hence the introduction of some bias in the reported health seeking findings | | To rule out biases on data regarding health seeking practices, ARISES study recruited all of its participants from the communities. |
|  | Finally, while the SIREN study proposed to use its findings to enhance stroke prevention, and community participation... | | ARISES study has already taken practical measures towards this outcome. It designed and launched the stroke information surveillance system (SISS), the first of its kind m-health system demonstrates practical efforts of the ARISES study towards stroke prevention, capacity building and community participation. The SISS, which incorporated findings from both the SIREN and ARISES qualitative studies, aims to correct stroke myths/misconceptions, appropriate stroke risk factor education and prevention, and to elicit timely referral to health facilities for management and reduction of the occurrence of disabilities across communities. This system and its components ~~is~~ are not only targeted for communities, but also to all alternative/complementary providers/healers and orthodox/modern medicine/health care providers across both urban and rural communities, thus strengthening capacity of HCP towards stroke prevention, care and recovery. The ARISES study findings also highlights recommendations on how to ensure the sustainability of the SISS with the collaborative efforts from the academia, community and the government. |

5. Please ensure that you refer to Figure 1 in your text as, if accepted, production will need this reference to link the reader to the figure. *This has been addressed accordingly*

6. Please change the highlighted text in your manuscript with black text. *This has been addressed accordingly*

Sincerely,

Prof. Mayowa O. Owolabi, *DrM, MD, FAAN, FANA, FRCP, FAMedS, FAAS, FAS*

Center for Genomics and Precision Medicine,

College of Medicine, University of Ibadan, Ibadan.

Tel: +234 802 077 5595; Email: [mayowaowolabi@yahoo.com](mailto:mayowaowolabi@yahoo.com)

ORCID #: https://orcid.org/0000-0003-1146-3070

We look forward to positive consideration and publication of this manuscript.
