# Supplementary material for: Community voices: Exploring beliefs, attitudes, practices and recommendations for improving stroke prevention and stroke care in rural and urban communities in Nigeria
Source: PLoS One. 2026 Feb 2;21(2):e0322157. doi: 10.1371/journal.pone.0322157 (PMC12863480; doi:10.1371/journal.pone.0322157)
Supplement: Attachment — Submitted filename: ARISES_PLOS_ONE_MANUSCRIPT_24092025_RESPONSE_TO_REVIEWERS.docx [file pone.0322157.s006.docx]

----- Forwarded message -----

From: "PLOS ONE" <[em@editorialmanager.com](mailto:em@editorialmanager.com)>

To: "Mayowa Ojo Owolabi" <[mayowaowolabi@yahoo.com](mailto:mayowaowolabi@yahoo.com)>

Cc:

Sent: Wed, 24 Sept 2025 at 3:23 am

Subject: Your Submission: PONE-D-24-25604R2 - [EMID:44725e04dd36a0fd]

PONE-D-24-25604R2

Community Voices: Exploring Beliefs, Attitudes, Practices and Recommendations for Improving Stroke Prevention and Stroke Care in Rural and Urban Communities in Nigeria.

PLOS ONE

Dear Dr Owolabi,

Thank you for submitting your manuscript to PLOS ONE.

We emailed you on July 16, 2025; below there is a detailed copy of this email and if you are having difficulties completing these amendments, do not hesitate in coming back to me. Please also ensure that you send back the original submission and do not submit a new one.

Please be aware that if we do not receive your submission or hear from you within 7 days, your manuscript will be withdrawn from the PLOS ONE system.

Kind regards,

Maidelyn R. Peregrin

PLOS ONE

Date:    Jul 16 2025 09:24AM

To:    "Mayowa Ojo Owolabi" [mayowaowolabi@yahoo.com](mailto:mayowaowolabi@yahoo.com)

From:    "PLOS ONE" [plosone@plos.org](mailto:plosone@plos.org)

Subject:    PLOS ONE: Edits requested on your submission PONE-D-24-25604R2

=============================================

PONE-D-24-25604R2

Community Voices: Exploring Beliefs, Attitudes, Practices and Recommendations for Improving Stroke Prevention and Stroke Care in Rural and Urban Communities in Nigeria.

Dr Mayowa Ojo Owolabi

Dear Dr. Owolabi,

We've checked your submission and before we can proceed, we need you to address the following issues:

1. Please include a copy of Table 1 and 2, which you refer to in your text on page 10.

We've returned your manuscript to your account. Please resolve these issues and resubmit your manuscript within 21 days. If you need more time, please email the journal office at [plosone@plos.org.](mailto:plosone@plos.org.) We are happy to grant extensions of up to one month past this due date. If we do not hear from you within 21 days, we will withdraw your manuscript.

Please log on to PLOS Editorial Manager at <https://www.editorialmanager.com/pone/> to access your manuscript. You will find your manuscript in the 'Submissions Sent Back to Author' link under the New Submissions menu. Be sure to remove your previous manuscript file if you are uploading a new file in response to these requests. After you've made the changes requested above, please be sure to view and approve the revised PDF after rebuilding the PDF to complete the resubmission process.

We are requesting these changes to comply with the PLOS ONE submission guidelines (<https://journals.plos.org/plosone/s/submission-guidelines>). Please note that we won't send your manuscript for review until you have resolved the above requests.

Thank you for submitting your work to PLOS ONE and supporting our mission of Open Science.

Kind regards,

Maidelyn R. Peregrin

PLOS ONE

**TABLE SHOWING RESPONSES TO REVIEWERS COMMENTS**

| **REVIEWER’S COMMENTS** | **AUTHORS’ RESPONSES (IN RED)** |
| --- | --- |
| Please include a copy of Table 1 and 2, which you refer to in your text on page 10. | Thank you for your comment which is well acknowledged.  Kindly note that the Tables 1 and 2 referred to under page 10 was a typo error. The participants details mentioned under page 10 have now been presented as descriptives (see previous cover letter and table highlighting all comments below). Hence, appropriate corrections and table numberings have been made on the clean and tracked version of the manuscript. |
