# Supplementary material for: Community voices: Exploring beliefs, attitudes, practices and recommendations for improving stroke prevention and stroke care in rural and urban communities in Nigeria
Source: PLoS One. 2026 Feb 2;21(2):e0322157. doi: 10.1371/journal.pone.0322157 (PMC12863480; doi:10.1371/journal.pone.0322157)
Supplement: Attachment — Submitted filename: ARISES_PLOS_ONE_MANUSCRIPT_04122025_RESPONSE_TO_REVIEWERS.docx [file pone.0322157.s007.docx]

**From:** "PLOS ONE" <[em@editorialmanager.com](mailto:em@editorialmanager.com)>

**To:** "Mayowa Ojo Owolabi" <[mayowaowolabi@yahoo.com](mailto:mayowaowolabi@yahoo.com)>

**Cc:**

**Sent:** Wed, 22 Oct 2025 at 7:38 am

**Subject:** PLOS ONE Decision: Revision required [PONE-D-24-25604R2] - [EMID:24f4ad6229286c25]

PONE-D-24-25604R2

Community Voices: Exploring Beliefs, Attitudes, Practices and Recommendations for Improving Stroke Prevention and Stroke Care in Rural and Urban Communities in Nigeria.

PLOS ONE

Dear Dr. Owolabi,

Thank you for submitting your manuscript to PLOS ONE. After careful consideration, we feel that it has merit but does not fully meet PLOS ONE’s publication criteria as it currently stands. Therefore, we invite you to submit a revised version of the manuscript that addresses the points raised during the review process. First, we apologize again for the delay you have experienced in receiving this decision. Please review the comments from the reviewers. The main issue to address is a possible decrease in the length of the Results section, as suggested by reviewer 4. This will improve the readability of your manuscript.

Please submit your revised manuscript by Dec 05 2025 11:59PM. If you will need more time than this to complete your revisions, please reply to this message or contact the journal office at [plosone@plos.org](mailto:plosone@plos.org). When you're ready to submit your revision, log on to <https://www.editorialmanager.com/pone/> and select the 'Submissions Needing Revision' folder to locate your manuscript file.

Please include the following items when submitting your revised manuscript:

- A rebuttal letter that responds to each point raised by the academic editor and reviewer(s). You should upload this letter as a separate file labeled 'Response to Reviewers'.
- A marked-up copy of your manuscript that highlights changes made to the original version. You should upload this as a separate file labeled 'Revised Manuscript with Track Changes'.
- An unmarked version of your revised paper without tracked changes. You should upload this as a separate file labeled 'Manuscript'.

If you would like to make changes to your financial disclosure, please include your updated statement in your cover letter. Guidelines for resubmitting your figure files are available below the reviewer comments at the end of this letter.

If applicable, we recommend that you deposit your laboratory protocols in [protocols.io](http://protocols.io/) to enhance the reproducibility of your results. Protocols.io assigns your protocol its own identifier (DOI) so that it can be cited independently in the future. For instructions see: [https://journals.plos.org/plosone/s/submission-guidelines#loc-laboratory-protocols](https://track.editorialmanager.com/CL0/https:%2F%2Fjournals.plos.org%2Fplosone%2Fs%2Fsubmission-guidelines%23loc-laboratory-protocols/1/010f019a0a6d583a-bf7b8bb7-c1ab-48e3-9c16-2a48adb754d8-000000/HHoOCn5TLegl2azLOxoZpSAaqGXAdFDbwQuMR1r0OFQ=234). Additionally, PLOS ONE offers an option for publishing peer-reviewed Lab Protocol articles, which describe protocols hosted on [protocols.io](http://protocols.io/). Read more information on sharing protocols at [https://plos.org/protocols?utm_medium=editorial-email&utm_source=authorletters&utm_campaign=protocols](https://track.editorialmanager.com/CL0/https:%2F%2Fplos.org%2Fprotocols%3Futm_medium=editorial-email%26utm_source=authorletters%26utm_campaign=protocols/1/010f019a0a6d583a-bf7b8bb7-c1ab-48e3-9c16-2a48adb754d8-000000/xRauS_BUXslBbT8ZLD6NRnVvvlzdQ14LB8X80jM9Ptw=234).

We look forward to receiving your revised manuscript.

Kind regards,

Sarah Jose, Ph.D.
Staff Editor
PLOS ONE

**TABLE SHOWING RESPONSES TO REVIEWERS COMMENTS**

| **COMMENTS** | **AUTHORS’ RESPONSES (IN RED)** |
| --- | --- |
| **Journal requirements note**  If the reviewer comments include a recommendation to cite specific previously published works, please review and evaluate these publications to determine whether they are relevant and should be cited. There is no requirement to cite these works unless the editor has indicated otherwise. Please review your reference list to ensure that it is complete and correct. If you have cited papers that have been retracted, please include the rationale for doing so in the manuscript text, or remove these references and replace them with relevant current references. Any changes to the reference list should be mentioned in the rebuttal letter that accompanies your revised manuscript. If you need to cite a retracted article, indicate the article’s retracted status in the References list and also include a citation and full reference for the retraction notice. | Thank you for your comment which is well acknowledged.  Kindly note that all references cited in this manuscript are relevant. Also, none of our references have been retracted to the best of our knowledge |
| **Comments from academic editor** | None |
| **Reviewer’s comments** |  |
| 1. If the authors have adequately addressed your comments raised in a previous round of review and you feel that this manuscript is now acceptable for publication, you may indicate that here to bypass the “Comments to the Author” section, enter your conflict of interest statement in the “Confidential to Editor” section, and submit your "Accept" recommendation.  Reviewer #2: All comments have been addressed  Reviewer #4: (No Response) | Thank you for your response which is well received. |
| 2. Is the manuscript technically sound, and do the data support the conclusions? The manuscript must describe a technically sound piece of scientific research with data that supports the conclusions. Experiments must have been conducted rigorously, with appropriate controls, replication, and sample sizes. The conclusions must be drawn appropriately based on the data presented.  Reviewer #2: Yes  Reviewer #4: Yes | Thank you for your response which is well received. |
| 3. Has the statistical analysis been performed appropriately and rigorously?  Reviewer #2: N/A  Reviewer #4: N/A | Thank you for your response which is well received. |
| 4. Have the authors made all data underlying the findings in their manuscript fully available? The [PLOS Data policy](http://track.editorialmanager.com/CL0/http:%2F%2Fwww.plosone.org%2Fstatic%2Fpolicies.action%23sharing/1/010f019a0a6d583a-bf7b8bb7-c1ab-48e3-9c16-2a48adb754d8-000000/5MsoCrVM5OwLlazPrj129pmYfO12FuN4FpAhIMYqhI0=234) requires authors to make all data underlying the findings described in their manuscript fully available without restriction, with rare exception (please refer to the Data Availability Statement in the manuscript PDF file). The data should be provided as part of the manuscript or its supporting information or deposited to a public repository. For example, in addition to summary statistics, the data points behind means, medians and variance measures should be available. If there are restrictions on publicly sharing data—e.g. participant privacy or use of data from a third party—those must be specified.  Reviewer #2: Yes  Reviewer #4: Yes | Thank you for your response which is well received. |
| 5. Is the manuscript presented in an intelligible fashion and written in standard English? PLOS ONE does not copyedit accepted manuscripts, so the language in submitted articles must be clear, correct, and unambiguous. Any typographical or grammatical errors should be corrected at revision, so please note any specific errors here.  Reviewer #2: Yes  Reviewer #4: Yes | Thank you for your response which is well received. |
| Review Comments to the Author Please use the space provided to explain your answers to the questions above. You may also include additional comments for the author, including concerns about dual publication, research ethics, or publication ethics. (Please upload your review as an attachment if it exceeds 20,000 characters). | Not applicable |
| Reviewer #2: The authors have addressed all my concerns. By eliminating the tables 3 and 4, an explanation of these findings makes better reading than before. The methods section has also been improved with more detailed explanation of what they set out to achieve. The discussion also flows in tandem with the results. | Thank you for your response which is well received. |
| Reviewer #4: The authors did a nice job of re-working this manuscript, notably expanding on the results and making tables 3&4 into supplementary data. Thank you for addressing the recommended changes. | Thank you for your response which is well received. |
| My main recommendation for revisions is to condense the results section as it is extensively long and remove many of the quotes provided. In qualitative research, a quote should only be provided when it illustrates a point you are explaining. Instead, the authors appear to provide quotes as a way to "back up" their results analysis. This is not needed. Please summarize the main findings and only provide quotes when it helps illustrate a particular point. | Thank you for your comments.  Overall, the qualitative findings section have been condensed from ten (page 15-25) pages to six (page 15-20) pages. Also supporting statements/quotations have also been revised to retain most relevant ones.  Under the KII findings, all sub-themes under the theme ‘risk susceptibility’ found in pages 14-15, paragraph 1-4 have been condensed into a single paragraph. The sub-themes under the theme, ‘ risk severity’ have been condensed into a paragraph and can be found in page 15-16, paragraph 2. The same approach have been applied to other themes which are now condensed to one paragraph each as found in pages 16– 17.  The same approach was applied to the FGD findings. These findings which occupied 4 pages, have now been condensed to two pages under pages 18-20. |
| Page 16: "bagged a university degree" -- this word is slang. Please reconsider your word choice (e.g., received, obtained, earned). | Thank you for your response. This has been revised in the main document under page 14 paragraphs 2. |
|  |  |
